# Supplementary material for: Data-driven discovery of canonical large-scale brain dynamics
Source: Cereb Cortex Commun. 2022 Nov 2;3(4):tgac045. doi: 10.1093/texcom/tgac045 (PMC9721525; doi:10.1093/texcom/tgac045)
Supplement: supplementary_material_tgac045 [file supplementary_material_tgac045.pdf]

Supplementary material of "Data-driven discovery of canonical large-scale brain dynamics"

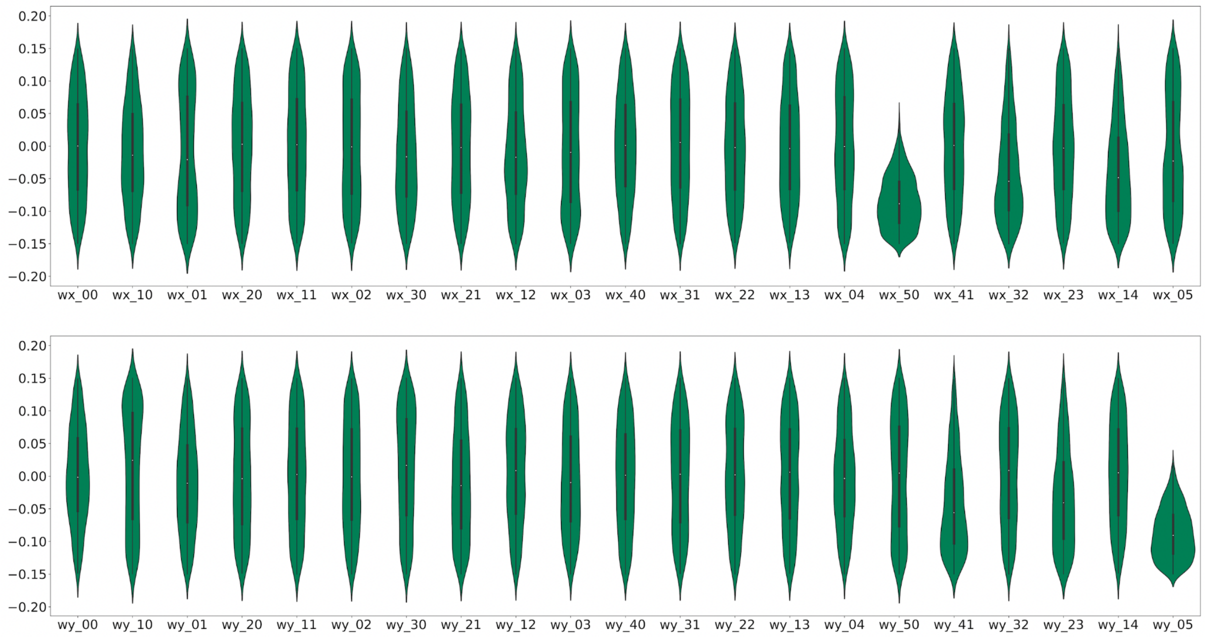

Supplementary Figure 1. Violin plots showing the distribution of optimal parameters found across all iterations of the optimization procedure. Parameters wx_ij / wy_ij correspond to the term $x^i y^j$ in the equations for $\frac{dx}{dt}$ / $\frac{dy}{dt}$, respectively.

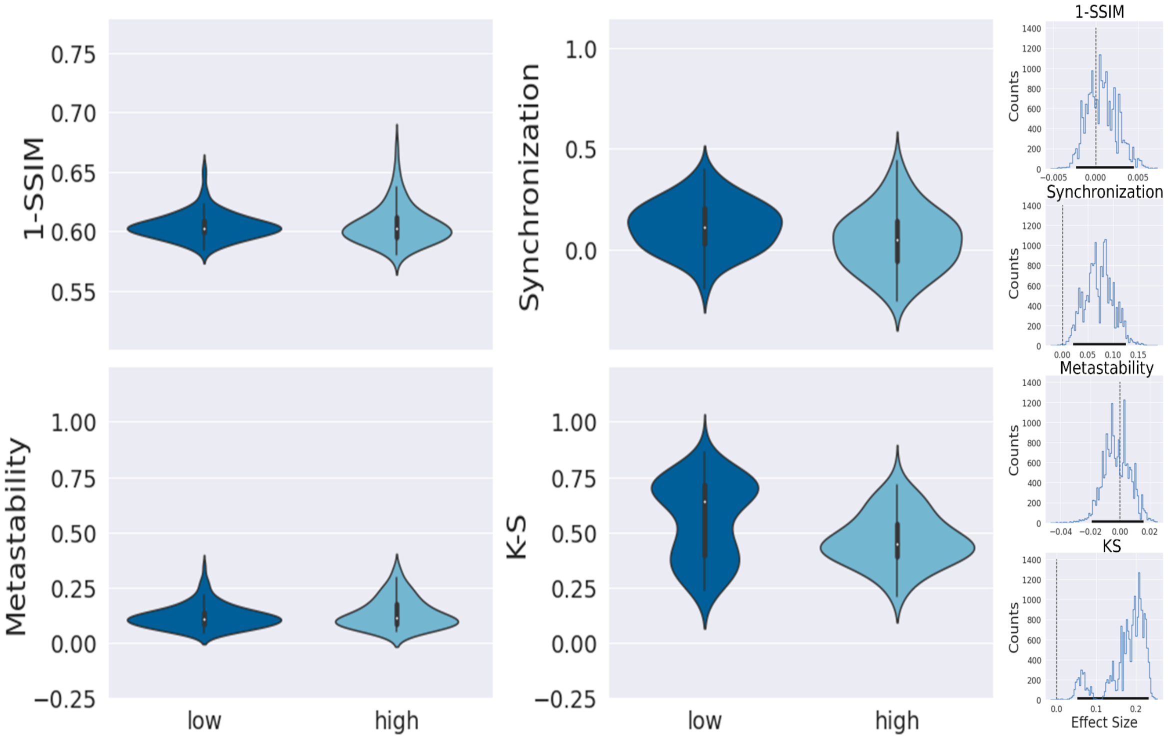

Supplementary Figure 2. Effect of the distance to the bifurcation (unstable spiral) on goodness of fit metrics. The violin plots summarize the distribution of the performance metrics for the 20 solutions closest to the bifurcation ('low') and the ones farther away from the bifurcation ('high'). The right panel shows the distribution of effect sizes for the difference in the performance metrics obtained using bootstrap. The vertical line indicates zero, i.e., null effect size, while the 95% confidence intervals are indicated using thick black lines in the x-axis. Only synchronization and KS of FCD showed statistically significant differences between both cases.
